# Identification and characterization of the elusive protein backbone of the immuno-dominant and species-specific Em2(G11) metacestode antigen of *Echinococcus multilocularis*

**DOI:** 10.3389/fpara.2025.1540215

**Published:** 2025-03-11

**Authors:** Philipp A. Kronenberg, Teivi Laurimäe, Michael Reinehr, Ansgar Deibel, Sina Hasler, Peter Gehrig, Achim Weber, Peter Deplazes, Ramon M. Eichenberger

**Affiliations:** ^1^ Institute of Parasitology, Vetsuisse and Medical Faculty, University of Zurich, Zurich, Switzerland; ^2^ Medical Micro- and Molecular Biology, Institute of Chemistry and Biotechnology, Zurich University of Applied Sciences (ZHAW), Wädenswil, Switzerland; ^3^ Department of Zoology, Institute of Ecology and Earth Sciences, University of Tartu, Tartu, Estonia; ^4^ Department of Pathology and Molecular Pathology, University of Zurich and University Hospital Zurich, Zurich, Switzerland; ^5^ Institute of Pathology, Hegau-Bodensee Clinic, Gesundheitsverband Landkreis Konstanz (GLKN), Singen, Germany; ^6^ Departement for Gastroenterology and Hepatology, University Hospital Zurich, Zurich, Switzerland; ^7^ Functional Genomics Center Zurich, University of Zurich and Federal Institute of Technology (ETH) Zurich, Zurich, Switzerland

**Keywords:** Em2, mAbEm2G11, monoclonal antibody, proteomics, LC-MS, deglycosylation, mucin, IHC

## Abstract

Alveolar echinococcosis (AE) caused by *Echinococcus multilocularis*, is a severe zoonotic disease in humans. One of the major metacestode antigens of *E. multilocularis* is the Em2 or Em2(G11) native purified antigen. The Em2 antigen is used for the serological and histopathological diagnosis of AE in humans and plays an important role in parasite–host interactions. As the Em2(G11) antigen is a mucin-type and glycosylated protein, the protein backbone has not been identified yet. We have targeted the protein backbone identification through mass spectrometry (LC-MS/MS) analysis of the Em2(G11) antigen. As a result, we evidenced that the Em2(G11) antigen consists of 33 unique protein candidates of which the most abundant was ‘’EmuJ_001105600.1’’. This protein (889 amino acids) had 427 predicted glycosylation sites. Amino acid composition comparison was in agreement with earlier studies and further confirmed the candidate of interest as the most likely Em2(G11) protein backbone. NCBI BLAST revealed no other known protein homologues in related *Echinococcus* species nor helminths. After successfully producing this protein recombinantly (Em2rec), a monoclonal antibody (mAbEm2rec) was raised against it. Immunohistochemical stainings of liver tissue sections of AE patients showed that the mAbEm2rec reacts specifically with *E. multilocularis* antigens solely after deglycosylation with an *O*-glycosidase cocktail. Similarly, in ELISA, the mAbEm2rec recognized the recombinant and native antigens of *E. multilocularis* after deglycosylation. These results reveal the nature of this highly glycosylated and specific protein, where mucins are covering the proteomic backbone. For antibody detection in human patients, the native Em2(G11) antigen was superior compared to the Em2rec antigen, indicating the importance of glycosylated epitopes in this immuno-dominant antigen. Of note is the second most abundant protein in the Em2(G11) antigen, namely phosphoenolpyruvate carboxykinase (PEPCK; EmuJ_000292700.1). PEPCK is known to play an important part in the metabolic pathway of gluconeogenesis in *E. multilocularis*. However, whether this co-eluted protein has any functional importance in the parasite-host interplay of nutrients, growth, and diagnostic significance, is not explored. By combining various approaches, we were able to uncover and confirm the protein backbone of the diagnostic Em2(G11) antigen of *E. multilocularis*.

## 1 Introduction

Alveolar echinococcosis (AE) is a severe parasitic disease. It is caused by the larval stage (metacestode) of the fox tapeworm *Echinococcus multilocularis* (Eckert and Deplazes, 2004). Due to its invasive and carcinoma-like growth in humans, it is regarded as one of the most dangerous zoonotic infections. The disease is of significant medical and public health importance due to its high morbidity and mortality if left untreated (Kern et al., 2017). It is endemic in certain regions of the Northern Hemisphere, including parts of Central Europe, Asia, and North America, where it poses a considerable burden on public health (Deplazes et al., 2017).

By primarily infecting the liver, the metacestode stage is characterized by fluid-filled infiltrative multivesicular structures that comprise an outer acellular laminated layer (LL) and an inner germinal layer (GL) (Eckert and Deplazes, 2004). The LL, with its heavily glycosylated structure, is thought to play a pivotal role in acting as both, a physical and an immunological barrier from the host (Gottstein et al., 2017). Various molecules have been identified as important structural components of the LL (e.g., EmAP (Lawton et al., 1997), EmP2 (Ingold et al., 1998), Em492 (Walker et al., 2004), reviewed in Gottstein and Hemphill, 2008). A monoclonal antibody (mAbEm2G11) that specifically targets the Em2(G11) antigen has previously revealed that the Em2(G11) antigen is one of the key immunogenic components of the LL (Deplazes and Gottstein, 1991). The *E. multilocularis* specific Em2 (Gottstein et al., 1983) or Em2(G11) (Deplazes and Gottstein, 1991) antigen remain as one of the state-of-the-art diagnostic tools used for the specific serological diagnosis of AE (Gottstein et al., 2019, 1993; Kronenberg et al., 2022). In humans, the Em2(G11) antigen can be detected outside of the lesions in the surrounding tissue as well as in lymph nodes (Ricken et al., 2017), and is secreted toward the outside of cultured vesicles *in vitro* (Deplazes and Gottstein, 1991). Furthermore, the Em2(G11) antigen plays an important role in parasite–host interactions, by modulating T-cell responses (Gottstein et al., 2017).

Previous studies have suggested that the Em2 antigen is primarily composed of carbohydrate moieties (Gottstein et al., 1994). By using a mass spectrometric *O*-glycan-profiling method and further amino acid composition analysis, Hülsmeier et al. (2002) later provided further evidence that the structural nature of the affinity-purified Em2(G11) antigen is that of a mucin-type glycosylated protein (*O*-linked glycan) with an unexpected large proportion of proline (5.3–6.4 mol%) and threonine (7.2–13.6 mol%) residues and comprises the following carbohydrates: galactose, N-acetylgalactosamine, and N-acetylglucosamine. Another study aimed to evaluate the diagnostic performance of these carbohydrate moieties through chemical synthesis of oligosaccharides, ultimately concluding that the immunogenic components of the antigen are likely the glycans (Yamano et al., 2012).

However, to this day, the identification of the protein backbone of the heavily glycosylated Em2(G11) antigen has remained elusive. This could be attributed to a multitude of factors, among them that the nature of the post-translational modification of *O*-glycosylation hinders the complete removal of oligosaccharides from the protein backbone (He et al., 2024). This, in turn, is a prerequisite for efficient proteolytic (tryptic) digestion of the proteins for successful identification through liquid-chromatography mass spectrometry (LC-MS) analysis (Saldova and Wilkinson, 2020). The main objective of the current study was to identify and characterize the elusive protein backbone of the species-specific and immuno-dominant Em2(G11) antigen of *E. multilocularis*.

## 2 Materials and methods

### 2.1 Proteomics identification of the Em2(G11) antigen

#### 2.1.1 Antigen samples

Two biological replicates of the native affinity-purified Em2(G11) antigen were submitted to mass spectrometry (LC-MS/MS) analysis for protein backbone identification. One replicate was of European (German) origin (J2012 isolate), while the second replicate originated from Kyrgyzstan (AT17 isolate). Both Em2(G11) antigens were produced from *in vitro* cultivated vesicles without host tissue and affinity-purified with the monoclonal antibody mAbEm2G11 (Kronenberg et al., 2022).

#### 2.1.2 Sample preparation for LC-MS/MS

As evidenced in a previous study by Hülsmeier et al. (2002), the Em2(G11) antigen is an *O*-linked protein (mucin) with a highly glycosylated structure. In order to allow for more efficient enzyme digestion of the protein prior to the LC-MS analysis, the antigen samples were first deglycosylated using the Protein Deglycosylation Mix II (New England Biolabs, Ipswich, USA) according to the manufacturer’s protocol. The deglycosylation mix contained the following enzymes: PNGase F, *O*-Glycosidase, α2-3,6,8,9 Neuraminidase A, β1-4 Galactosidase S, β-N-acetylhexosaminidase-f. Furthermore, to facilitate a more comprehensive removal of the sugars, two additional enzymes; β1-3 Galactosidase and α1-3,4,6 Galactosidase were added to the deglycosylation reaction according to the manufacturer’s instructions (New England Biolabs; Ipswich, USA). Following the deglycosylation reaction, both the European and Asian Em2(G11) antigen samples were split into two biochemical replicates, resulting in a total of four antigen replicates for the subsequent LC-MS analysis. Protein digestion for LC-MS/MS was performed using the filter-aided sample preparation method (FASP; (Wiśniewski, 2018)). In brief, lysis buffer containing 4% SDS and 0.1 M DTT in 0.1 M Tris-HCl, pH 8.2 was added to 25 µl of antigen sample in a 1:5 sample to buffer ratio. Next, 200 µl of 8 M urea/0.1 M Tris-HCl, pH 8.2 was added per 30 µl of sample and lysis buffer solution, followed by transferring the resulting mixture to a Microcon 30 (Merck, Germany) filter unit and centrifugation at 14 000 x g at RT for 25 min, discarding the flow-through. The process was repeated until all the sample was loaded onto the filter. The concentrate remaining on the filter was then diluted by adding 200 µl of 8 M urea in 0.1 M Tris-HCl, pH 8.2 and centrifuged at 14 000 x g at RT for 25 min. Subsequently, alkylation of free cysteines was achieved by adding 0.05 M IAA, mixing at 600 rpm for 1 min and further incubated on the bench another 5 min, followed by centrifugation as described above. The samples were then washed with 100 µl of 8 M urea in 0.1 M Tris-HCl, pH 8.2 and centrifuged, with the wash step repeated a total of three times. Two additional washing steps were done by adding 100 µl of 0.5 M NaCl to the filter and centrifugation as described above, followed by three additional washing steps with 0.05 M TEAB. The concentrate was then subjected to proteolytic overnight digestion at RT in a wet cell by adding 0.05 M TEAB and sequencing grade modified trypsin (Promega, USA) in a 1:50 trypsin to protein ratio (assumed protein weight 25 µg). The following day the digested samples were centrifuged at 14 000 x g at RT for 20 min and the pH adjusted by adding 5% trifluoroacetic acid (TFA, end concentration 0.5%), followed by drying the tryptic peptides in a SpeedVac concentrator. The samples were then resuspended in 30 µl solution of 3% acetonitrile (ACN) and 0.1% TFA and desalted using ZipTip® C18 pipette tips (Merck, Germany).

#### 2.1.3 LC-MS/MS

Data dependent analysis (DDA) was run on an Orbitrap Fusion LUMOS Tribrid mass spectrometer (ThermoFisher Scientific, USA) operated in conjunction with nanoAcquity UPLC M-class system and a symmetry C18 trap column and HSS T3 analytical column (Waters, USA). Injection volume per sample was 4 µl. The peptides were eluted with an ACN/water linear gradient at a 0.3 µl/min flowrate from 5% to 36% solvent B over 60 min with solvent A containing 0.1% FA in water and solvent B 0.1 FA in ACN. Full-scan MS spectra (MS1) in profile mode was acquired in the scan range of 300–2000 m/z at 120,000 resolution, with the precursor automated gain control (AGC) set to 500,000 and a maximum injection time (maxIT) of 50 ms. Precursors with +2 to +7 charge states and intensities over 5000 were selected for tandem mass spectrometry (MS2). Every full MS1 scan was followed by DDA scans recorded in centroid mode. The AGC target was set to 5’000 and the maxIT to 80 ms. Isolated precursors were fragmented with higher-energy collisional dissociation (HCD) at a normalized collision energy (NCE) of 35%. Fixed first mass was set to 140 m/z. Exclusion list for precursor masses already selected for MS2 measurement was set at 25 s with the exclusion window set at 10 ppm.

#### 2.1.4 Proteome data analysis

Available *Echinococcus* spp. reference proteomes were obtained from the UniProt and WormBase Parasites databases (version WBPS13). Proteomes of *E. multilocularis* (UP000017246; Tsai et al., 2013), and the closely related taxa of *E. granulosus* sensu *stricto* G1 (UP000492820; Tsai et al., 2013) and *E. canadensis* cluster G7 (PRJEB8992; Maldonado et al., 2017) were included for the protein identification. An in-house (Functional Genomics Centre Zurich) common contaminants database was also included in the protein identification searches. The Mascot (Matrix Science, UK) search engine was utilized to obtain initial protein identifications, with the following settings: trypsin as the proteolytic enzyme, allowing up to a maximum of two missed cleavages, carbamidomethylation of cysteine as a fixed and oxidation of methionine as a variable modification. Subsequently, further analysis and filtering of identified proteins was performed in Scaffold (Proteome Software, Inc), with protein threshold set to 95%, minimum peptide identifications to two, and peptide threshold set to 1.0% false discovery rate (FDR). For further filtering of the protein candidates of interest in Scaffold, only proteins that were identified across all four replicates with high confidence were selected for subsequent analysis. For the remaining candidates, the amino acid composition was estimated using ProtParam by Expasy (https://web.expasy.org/protparam/; Wilkins et al., 1999) and candidates with the closest similarity to the amino acid composition of Em2(G11) as described in Hülsmeier et al. (2002) with high proline and threonine concentrations were selected for recombinant protein production. Additionally, verification based on the assumption of the protein-backbone of the major immunogenic component of the affinity-purified Em2(G11) antigen being a mucin-type glycosylated protein included the presence of O-linked glycosylation-sites of the significantly abundant proteins. This was analyzed by the NetOGlyc 4.0 server with a threshold by the confidence score of >0.95 (https://services.healthtech.dtu.dk/services/NetOGlyc-4.0/) (Steentoft et al., 2013). Relative protein abundance by label-free quantification (LFQ) of the initial affinity-purified Em2(G11) proteins was performed in MaxQuant (version 2.3.1.0; Cox and Mann, 2008). The mass spectrometry proteomics data have been deposited in the ProteomeXchange Consortium via the PRIDE partner repository with the dataset identifier PXD056760 and https://doi.org/10.6019/PXD056760.

### 2.2 Validation of the Em2(G11) protein candidates

#### 2.2.1 Recombinant production of the most abundant Em2(G11) protein hit

Following filtering of the mass spectrometry results in Scaffold, a recombinant version of the best candidate (gene accession number EmuJ_001105600.1) was produced. Due to a significantly high content of repetitive sequences, the gene was codon optimized for expression in *E. coli*, commercially synthesized and cloned into the pET-32α (+) expression vector at the KpnI/NotI restriction sites (GenScript, USA), including a C-terminal poly-His affinity tag. Because of a potential interfering reaction in further approaches, the N-terminal thioredoxin (TRX) tail, thrombin (thr) cleavage site and the S-tag were exchanged with a commercially synthesized 28-bases nonsense fragment (Microsynth, Switzerland) at the XbaI/KpnI restriction sites, while maintaining the ribosome binding site, having the methionine start-codon (ATG) at the beginning of the gene candidate (**Supplementary Material**). The modified plasmid was chemically transformed by heat-shock into competent *E. coli* BL21 and screened for successful vector-uptake by T7-PCR and sequencing (Microsynth, Switzerland). The protein (Em2rec) was expressed after induction of 0.5 mM IPTG for 6 h at 26°C. After lysis and freezing, the soluble protein fractions released from the cells were filtered through a 0.45 µm filter and purified by Immobilized Metal Affinity Chromatography with a 1 mL His-Trap HP column (GE29-0510-21, Cytiva, USA) according to manufacturer’s instructions on an Äkta pure M1 FPLC (Cytiva, USA) at a flow rate of 1.0 ml/min and eluted by an increasing concentration of imidazole (40–500 mM). Eluted fractions that contained protein were pooled, buffer exchanged to phosphate buffered saline (PBS) and concentrated using a 10 kDa MWCO Amicon® Ultra centrifugation filter (UFC8010, Merck, Germany). The identity of the 92-kDa protein was confirmed by SDS-PAGE and Western blot using an anti-His monoclonal antibody (MA1-21315-BTIN, ThermoFisher Scientific, USA). The protein concentration was determined by Pierce BCA Protein Assay (23225, ThermoFisher Scientific, USA) and the protein was stored at −78°C until further use.

#### 2.2.2 Monoclonal antibodies and ELISA

A monoclonal antibody (mAb) against the Em2rec protein was generated for further identification of the epitope on protein level in ELISA and human liver sections, based on a modified protocol (de St. Groth and Scheidegger, 1980) according to Kronenberg et al. (2023). IgG1 isotype of the selected mAbEm2rec was determined by a mouse mAb isotyping ELISA according to the manufacturer’s instructions (ISO2-1KT, Sigma-Aldrich, USA). The final mAbEm2rec was screened by ELISA against the recombinant Em2rec protein and a crude *E. coli* lysate, and deglycosylated fractions of the diagnostic Em2(G11) antigen, various native and deglycosylated antigens of *E. multilocularis*, *E. granulosus s.s.*, *Taenia solium*, *Fasciola hepatica. Strongyloides ratti*, *Ascaris lumbricoides*, *Onchocerca jakutensis*, and *Dirofilaria immitis* (**Table 1**). Furthermore, a mAb panel including control mAbs from a previous study was included in the analysis (**Supplementary Table S4**). ELISA, including the origin of all antigens and mAbs, was performed as described by Kronenberg et al. (2023). In brief, 10 µg of antigen dissolved in 20 µl of PBS was incubated at room temperature for 30 min with 2.5 µl of a Protein Deglycosylation Mix II (New England Biolabs, NEB; P6044S) and 2.5 µl “10x Buffer I” from Protein Deglycosylation Mix II. Subsequently, 1 µl of β1-3 Galactosidase (NEB; P0726S) and 1 µl α1-3,4,6 Galactosidase (NEB; P0747S) were added and incubated overnight at 37°C. The antigens were then coated in a concentration of 5 µg/ml coating buffer and tested by ELISA.

**Table 1:** Binding of mAbEm2G11 and mAbEm2rec to native and deglycosylated *Echinococcus* spp. and other helminth antigens in ELISA.

		mAbEm2G11 #	mAbEm2rec #	control mAbD.i36/1 #	serum mouse AE §#
Species, G = genotype	Origin and stage of antigens (Ag) #	IgG1	IgG1	IgG1	
*E. multilocularis*	recombinant, Em2rec Ag	–	++++	–	++++
*E. multilocularis*	affinity purified Em2(G11) Ag	++++	–	–	++++
*E. multilocularis*	affinity purified Em2(G11) Ag*	–	++++	–	++++
*E. multilocularis*	*in vitro*, vesicle somatic Ag	++++	–	–	++++
*E. multilocularis*	*in vitro*, vesicle somatic Ag*	++	++++	–	++++
*E. multilocularis*	*in vitro*, pure vesicle fluid Ag	–	–	–	+++
*E. multilocularis*	*in vitro*, pure vesicle fluid Ag*	–	–	–	++
*E. multilocularis*	*in vitro*, vesicles ESP Ag	+++	–	–	++++
*E. multilocularis*	*in vitro*, vesicles ESP Ag*	–	–	–	++++
*E. multilocularis*	gerbil, protoscolex crude Ag	–	–	–	++++
*E. multilocularis*	gerbil, protoscolex crude Ag*	–	–	–	++++
*E. granulosus*, G1-3	*in vitro*, vesicle somatic Ag	–	–	–	++++
*E. granulosus*, G1-3	*in vitro*, vesicle somatic Ag*	–	–	–	++++
*E. granulosus*, G1-3	*in vitro*, pure vesicle fluid Ag	–	–	–	+++
*E. granulosus*, G1-3	*in vitro*, pure vesicle fluid Ag*	–	–	–	++
*E. granulosus*, G1-3	*in vitro*, vesicles ESP Ag	–	–	–	+
*E. granulosus*, G1-3	*in vitro*, vesicles ESP Ag*	–	–	–	+
*E. granulosus*, G1-3	sheep, protoscolex crude Ag	–	–	–	++++
*E. granulosus*, G1-3	sheep, protoscolex crude Ag*	–	–	–	++++
*Taenia solium*	pig, metacestode crude Ag	–	–	–	+
*Taenia solium*	pig, metacestode crude Ag*	–	–	–	+
*Fasciola hepatica*	cattle, adult worm crude Ag	–	–	–	++
*Fasciola hepatica*	cattle, adult worm crude Ag*	–	–	–	++
*Strongyloides ratti*	rat, larval crude Ag	–	–	–	+
*Strongyloides ratti*	rat, larval crude Ag*	–	–	–	++
*Ascaris lumbricoides*	human, adult worm crude Ag	–	–	–	+
*Ascaris lumbricoides*	human, adult worm crude Ag*	–	–	–	+
*Onchocerca jakutensis*	deer, adult worm crude Ag	–	–	–	+++
*Onchocerca jakutensis*	deer, adult worm crude Ag*	–	–	–	+++
*Dirofilaria immitis*	dog, adult worm crude Ag	–	–	+++	+++
*Dirofilaria immitis*	dog, adult worm crude Ag*	–	–	+++	+++

ELISA OD: − (0.0–0.05) / + (0.05–0.25) / ++ (0.25–0.5) / +++ (0.5–1.0) / ++++ (>1.0) / *deglycosylated according to Material & Methods, paragraph 2.2.2. / §serum of mouse infected with 500 *E. multilocularis* eggs and progressive alveolar echinococcosis (AE) / #ELISA according to Kronenberg et al. (2023).

### 2.3 Antibody detection in human AE patients by ELISA

ELISA was performed with the new recombinantly produced protein backbone Em2rec and compared to the native purified Em2(G11) antigen. Therefore, serum samples of 60 confirmed AE patients and 68 uninfected blood donors from a previous study were selected (Kronenberg et al., 2022). The test evaluation was based on the same study and compared by ROC analysis (**Supplementary Tables S2, S3**).

### 2.4 Immunohistochemical stainings (IHC-S)

**Figure 1:**
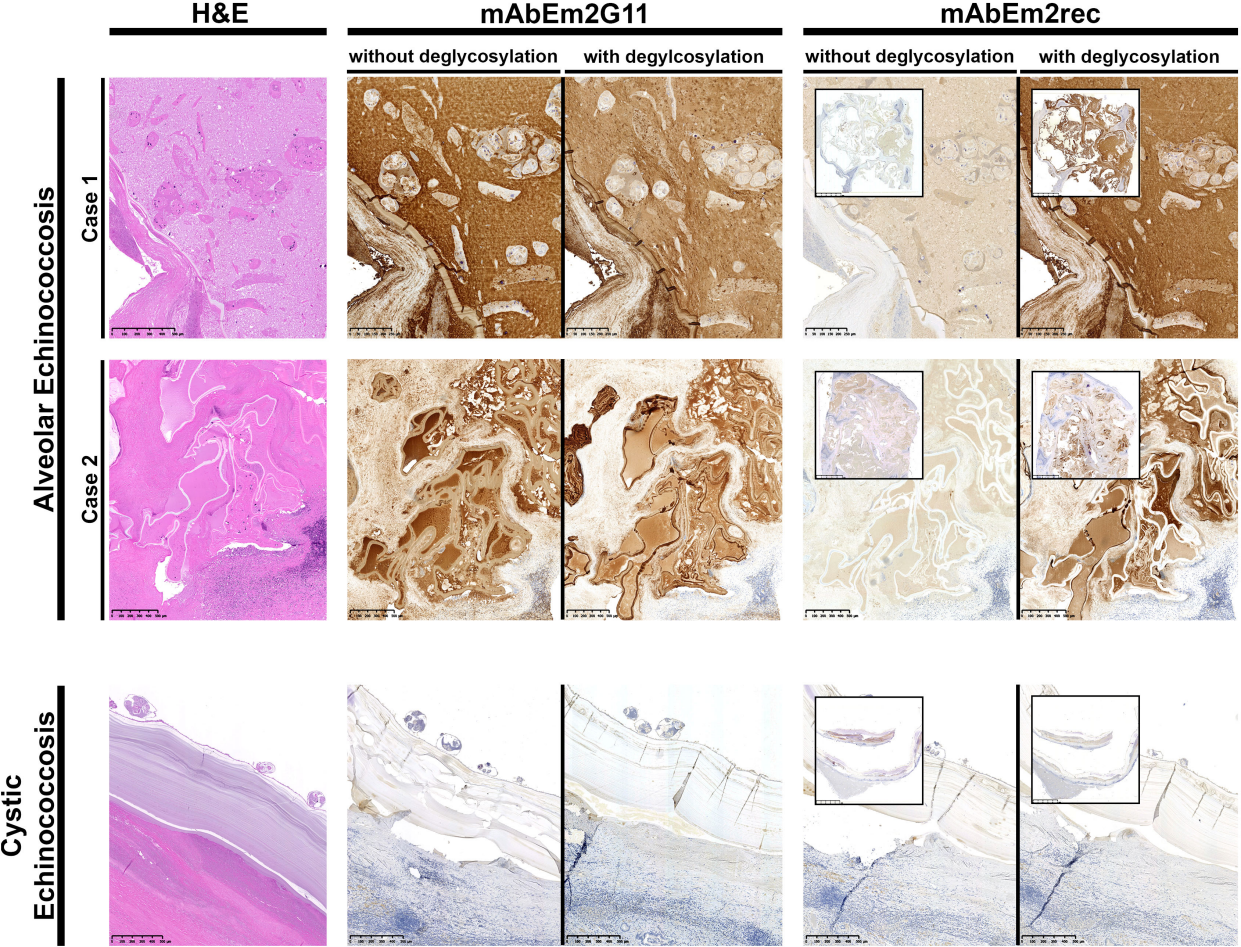
IHC-S with monoclonal antibodies mAbEm2G11 and mAbEm2rec on AE and CE liver sections without and with deglycosylation. Scale bars 250 µm (AE case 1.) 500µm (AE case 2. & CE case), insets 25mm.

For IHC-S, liver sections of two AE and one CE patient were selected from a previous study (Reinehr et al., 2020). In brief, FFPE fixation, deparaffinization and antigen retrieval was performed as described before (Reinehr et al., 2020; Robers et al., 2024). After the antigen retrieval, the slides were washed two times with PBS. Subsequently, the sections were incubated at 25°C for 30 min and then overnight at 37°C with 250 µl of an enzyme deglycosylation mix (190 µl PBS, 20 µl Protein Deglycosylation Mix II (New England Biolabs, NEB; P6044S), 25 µl “10x Buffer I” from Protein Deglycosylation Mix II, 5 µl β1-3 Galactosidase (NEB; P0726S), 5 µl α1-3,4,6 Galactosidase (NEB; P0747S), and 5 µl *O*-Glycoprotease (NEB; P0761S). Afterwards the slides were washed two times with PBS. The IHC-S with three monoclonal antibodies and hematoxylin and eosin (H&E) was performed as described earlier (Reinehr et al., 2020). The newly developed mAbEm2rec, directed against the protein backbone of the diagnostic Em2(G11) antigen, was compared to the widely used mAb Em2G11, which is directed against the native Em2(G11) antigen (**Figure 1**). Moreover, a *Dirofilaria* spp. specific monoclonal antibody (mAb D.i 36/1) was included in the analysis as a negative control IgG1 isotype (**Supplementary Figure S1**) (Joekel et al., 2017).

## 3 Results

### 3.1 LC-MS/MS identification of the Em2(G11) candidates

Across all four of the affinity-purified Em2(G11) antigen replicates, a total of 95 *E. multilocularis* derived proteins were detected, with 33 proteins confidently identified (FDR threshold >95%) across all four replicates (**Supplementary Table S1**). Of the 33 candidates, a single protein (UniProt gene accession number EmuJ_001105600.1) appeared to closely resemble the amino acid composition of the Em2(G11) antigen as described in Hülsmeier et al. (2002). Furthermore, the protein of interest featured a transmembrane domain, had an estimated molecular weight of 92 kDa, and was predicted to have 427 glycosylation sites, where the second most abundant protein (EmuJ_000292700.1; PEPCK) had no predicted O-linked glycosylation site. The Em2-candidate showed a high concentration of the amino acid proline (12.3 mol%) and threonine (33.3 mol%), respectively. Furthermore, this hit was the most abundant protein by label-free quantification (LFQ)-intensity values in the parasite proteomic dataset (**Supplementary Table S1**). The 92 kDa protein was subsequently produced recombinantly. Interestingly, no similar protein-homologue or gene was found in public NCBI databases for closely related *E. granulosus* sensu lato nor other helminths.

### 3.2 Diagnostic performance of native purified Em2(G11) and recombinant Em2rec antigen in ELISA

An overview of the diagnostic performance of the Em2rec and the native purified Em2(G11) antigen is given in **Supplementary Tables S2, S3**. To evaluate test performance, a serum panel of 60 confirmed human AE patients and 68 uninfected blood donors was included. Sensitivity and specificity were calculated based on ROC analysis and the highest Youden-Index. While native purified Em2(G11) antigen reached a sensitivity of 91.7% (95%CI: 80.9–96.9) and a specificity of 98.5% (95%CI: 91.0–99.9), the recombinant Em2rec antigen showed a sensitivity of 63.3% (95%CI: 49.8–75.1) and a specificity of 88.2% (95%CI: 77.6–94.4). No correlation between clinical data and test performances (by complementary testing) could be observed in the patient cohort.

### 3.3 Monoclonal antibodies targeting native and deglycosylated antigens of *Echinococcus* spp. and other helminths in ELISA

The mAbEm2G11 and mAbEm2rec were evaluated with a panel of 15 native and deglycosylated *Echinococcus* spp. and other helminth antigens (**Table 1**). While mAbEm2rec is directed against the recombinant protein backbone of the Em2(G11) antigen (Em2rec) and deglycosylated antigens of *E. multilocularis*, the mAbEm2G11 is targeting native antigens and shows no or only a weak signal after deglycosylation. Furthermore, mAbEm2G11 is targeting native secreted Em2 antigen *in vitro*, but no signal could be detected with mAbEm2rec to native nor deglycosylated excretory-secretory products. A panel of 4 more *Echinococcus* specific mAbs (mAbEm18, mAbAgB, mAbEmG3, and mAbEg2) was tested with the same conditions and the data is presented in **Supplementary Table S4**. A serum of a mouse infected with 500 *E. multilocularis* eggs and progressive alveolar echinococcosis served as a positive control for all applied antigens.

### 3.4 Immunohistochemical staining (IHC-S) of AE and CE liver sections

An overview of H&E staining and IHC-S with two monoclonal antibodies in two AE and one CE liver section is shown in **Figure 1**. While mAb Em2G11 is directed against the native Em2(G11) antigen, the newly developed mAbEm2rec is directed against the recombinant protein backbone of the same antigen. Whereas mAbEm2G11 shows a strong signal in AE liver metacestode sections with a slightly reduced staining in AE liver sections after deglycosylation treatment, the mAbEm2rec shows only very low, probably unspecific reactivity in untreated AE liver sections, but a strong signal in AE liver sections after deglycosylation treatment. No staining was observed with both monoclonal antibodies in a cystic echinococcosis (CE) liver section. As a control antibody, a mAb directed against *Dirofilaria* spp. antigens was included. No staining was observed in neither AE, nor in CE liver sections (**Supplementary Figure S1**).

## 4 Discussion

Over 40 years ago, the diagnostic Em2 antigen of *E. multilocularis* metacestodes was discovered by Gottstein et al. (1983). Affinity purification of a native crude metacestode antigen with rabbit polyclonal antibodies cross-absorbed against the metacestode of *E. granulosus s.l.*, resulted in a highly antigenic “Em2” flow-through fraction. The designated Em2 antigen allowed for the first time a specific serological identification of AE patients (Gottstein et al., 1983). This purification step was later optimized by using a monoclonal antibody (mAbEm2G11) directed against the Em2(G11) antigen (Deplazes and Gottstein, 1991). Both Em2 and Em2(G11) antigens exhibit the same test characteristics for serology in humans (Deplazes and Gottstein, 1991). Over the years, several studies aimed at characterizing the Em2 antigen (Hülsmeier et al., 2002; Yamano et al., 2012) and have shown the heavily glycosylated mucin-type structure of the Em2(G11) antigen. However, to this day, the protein backbone of the Em2(G11) antigen remained elusive.

In the current study, the Em2(G11) antigen was targeted by mass spectrometry (LC-MS/MS) analysis for protein backbone identification. Our study identified 33 potential protein backbone candidates, among which EmuJ_001105600.1 had the highest protein abundance. Furthermore, this candidate also appeared to have the greatest similarity in amino acid composition to that reported in a previous study (Hülsmeier et al., 2002), with a notably high content of proline and threonine (427 glycosylation sites). The genome of *E. multiloculari*s presents a second gene candidate with a comparable high content of proline and threonine (EmuJ_000938200). This smaller protein (33.6 kDa) with similar amino acid repeats however was excluded from the final list by stringent filtering. To confirm that the protein candidate of interest represents the Em2(G11) backbone, a mAb was produced against the recombinantly produced Em2rec protein (recombinant EmuJ_001105600.1). This mAbEm2rec recognized the recombinant construct Em2rec and the “native” Em2(G11), but only if the antigen was previously deglycosylated. In comparison, the original mAbEm2G11 solely targets the native Em2(G11) antigen and shows no signal in the recombinant backbone protein (**Table 1**). As both mAbs are highly specific for *E. multilocularis*, we could show that not only the glycosylated Em2(G11) antigen is species-specific, but also the protein backbone. No similar protein-homologue or gene was found in public NCBI databases searching for *E. granulosus s.l.* nor other helminth species. This indicates the species-specific nature of this protein and could play an important role in the unique pathology and host modulation of *E. multilocularis*.

Interestingly, the mAbEm2G11 targets native but not deglycosylated excretory-secretory products (ESP) of *E. multilocularis*, while mAbEm2rec does not recognize neither native nor deglycosylated ESP (**Table 1**). Several other studies have found the Em2 antigen to be present in the secreted fraction *in vitro* (Deplazes and Gottstein, 1991) and to some extent circulating in serum of human AE patients (Kronenberg et al., 2023), stained in human lymph nodes (Grimm et al., 2020) and detected in serum of dogs with AE (Staebler et al., 2006). This is indicative for a cellular processing and secretion of the carbohydrate-part. *O*-glycans and mucins have been described from various helminths (Hokke and van Diepen, 2017) and are a common finding in the parasite ESP. For example, three mucin-glycoproteins are secreted abundantly from the migratory larvae of the nematode *Toxocara canis* with a pivotal role in the immunoregulation of the host (Gems and Maizels, 1996; Loukas et al., 2000). Furthermore, in the model trematode *Echinostoma caproni* mucin-glycoprotein secretion via extracellular vesicles has been demonstrated without further functional information (Marcilla et al., 2012). However, a pathway of secretion of the carbohydrate-part has never been shown for helminths. The Em2(G11) *O*-glycan structure (carbohydrate-part of the mucin) is characterized by extended galactosylation (Hülsmeier et al., 2002). This pattern is also typical for glycolipids (glycosphingolipids) and discussed to be responsible for cross-reactions between platyhelminths. For example, similarly galactosylated sphingolipids have been described in the metacestode from the related cestode *Taenia crassiceps* (Dennis et al., 1992). The secretion of this immunodominant carbohydrate in *E. multilocularis* metacestodes, e.g. as glycolipid-conjugate via extracellular vesicles, and its role in the parasite–host interaction has to be further explored.

To verify the findings in parasite specimens from patients, immunohistochemical stainings similar to those applied in the routine clinico-pathological setting (Barth et al., 2012; Reinehr et al., 2020) were performed. The ELISA results could be replicated only partially by IHC-S. For both ELISA and IHC-S we have adapted a deglycosylation protocol from Wang et al (Wang et al., 2021), which depletes the parasite-specific glycosylated mucin-type epitope of the protein. However, the deglycosylation protocol in complex and relatively thick tissue slides has shown to be incomplete, reflected by the reduced but not absent staining pattern of mAbEm2G11 on deglycosylated AE liver sections. The mAbEm2G11 is staining the laminated layer, but not protoscolices of *E. multilocularis*, as well as spems (small particles of *E. multilocularis*) in the metacestode section. In this context, it is difficult to assess the function of spems connected to the Em2(G11) antigen in native and deglycosylated AE liver sections. Spems are localized outside of AE lesions and can be stained with mAbEm2G11 in nearby lymph nodes (Barth et al., 2012; Grimm et al., 2020; Reinehr et al., 2020; Ricken et al., 2017). However, with mAbEm2G11, spems show a reduced staining pattern after deglycosylation and are not completely absent. Nevertheless, as an additional verification of our gene candidate, the staining pattern could be reproduced with the mAbEm2rec in deglycosylated liver sections.

By a mass spectrometry analysis of the antibody-affinity purified diagnostic Em2(G11) antigen, a cocktail of 33 proteins was reliably detected. Although while not a pure antigen, it allows for an accurate differential diagnosis of patients with alveolar- and cystic echinococcosis, and other helminthic infections (Gottstein et al., 2019; Kronenberg et al., 2022). After recombinantly producing the protein candidate “EmuJ_001105600.1” as a potential protein backbone of the Em2(G11) antigen, we have evaluated it in ELISA. However, the diagnostic test characteristics (Se: 63.3%, Sp: 88.2%) could not reach the excellent test performance of native purified Em2(G11) antigen (Se: 91.7%, Sp: 98.5%). Therefore, no further testing was done regarding its cross-reactivity with other helminths. The immunogenic part from the diagnostic antigen fraction Em2(G11) is a mucin-type glycoprotein (Hülsmeier et al., 2002), where the carbohydrate moiety Galα1-4Galβ1-3GalNAc is the dominant epitope (Yamano et al., 2012). The presented approach cannot exclude the possibility of other mucin-type glycoproteins in the Em2(G11) antigen with a similar glycosylation pattern. Indeed, by predicting potential O-linked glycosylation sites of the 33 proteins in the proteomic dataset, another protein (EmuJ_000756700) with a high number (68) of predicted glycosylation sites was found (**Supplementary Table S1**). This “expressed conserved protein” has a domain of unknown function (DUF56734) which is found in various platyhelminths. Given the low abundance of this protein and the high specific test performances of the Em2(G11) diagnostics, we rule out a major role of this protein candidate in the specific serodiagnosis of AE.

Of note is the second most abundant protein in the Em2(G11) antigen (EmuJ_000292700.1), being annotated as phosphoenolpyruvate carboxykinase (PEPCK). Major sites of expression of mammalian PEPCK are kidney, adipocytes and in liver cells as a pivotal enzyme involved in the gluconeogenesis from non-carbohydrate sources (Stoffel et al., 1993) with the liver being also the primary location of *E. multilocularis* metacestodes. In *E. multilocularis*, PEPCK plays an important part in the metabolic pathway of gluconeogenesis (Ritler et al., 2019) and is especially upregulated in metacestodes being highly abundant in the proteome from crude metacestode preparations, cultured medium supernatant and the vesicular fluid (Huang et al., 2016; Müller et al., 2023). However, the role of the parasite PEPCK co-purified with the diagnostic Em2(G11) antigen is obscure: an involvement of PEPCK in the synthesis of the mucin-type carbohydrates is unlikely as there is no data of an involvement of PEPCK in the genesis of galactose, N-acetylgalactosamine, or N-acetylglucosamine, the main sugars of the *E. multilocularis* mucin carbohydrate composition (Hülsmeier et al., 2002). In other helminths, secreted PEPCK is known from the trematodes *Schistosoma mansoni* (Asahi et al., 2000) and *Fasciola hepatica* (Trelis et al., 2022), with a suspected uncharacterized moonlighting function in parasite adaptations to host energy environments and energy predation. Further, there are indications that in the *S. mansoni* egg-secreted antigens, PEPCK is involved in immune cell attraction resulting in a mixed Th1-Th2 response (Asahi et al., 2000). As the Em2(G11) antigen is also localized on the most outside layer of *E. multilocularis* metacestodes (Deplazes and Gottstein, 1991), PEPCK could be simply co-eluted without any diagnostic significance, whereas the role of PEPCK as a main part of the eluted diagnostic Em2(G11) antigen fraction and the potential involvement in parasite–host interactions remains unknown. Further studies on the diagnostic involvement of the different proteomic hits should be investigated.

## 5 Conclusions

By combining various approaches, we were able to uncover the protein backbone of the diagnostic Em2(G11) antigen of *E. multilocularis*. We confirmed that the main diagnostic epitope is a mucin, which covers the proteomic backbone. We could successfully produce the most abundant protein candidate recombinantly and further characterize its nature by monoclonal antibodies, ELISA and IHC-S. We could highlight, that the monoclonal antibody-affinity purified major diagnostic Em2(G11) antigen consists of at least 33 protein candidates, of which one species-specific protein (EmuJ_001105600.1) presents the most likely protein backbone of the diagnostic mucin-antigen. In addition, we discovered that phosphoenolpyruvate carboxykinase (PEPCK) is the second most abundant protein in the Em2(G11) antigen. As PEPCK serves in the metabolic pathway of gluconeogenesis, its function could be of high importance in the metabolomic parasite–host interplay, and points for further investigation.

## Data Availability

The original contributions presented in the study are publicly available. The mass spectrometry data have been deposited in the ProteomeXchange Consortium via the PRIDE partner repository with the dataset identifier PXD056760 and https://doi.org/10.6019/PXD056760.

## Supplementary material

Supplementary Figure 1 IHC-S with control monoclonal antibody mAb D.i 36/1 on deglycosylated and native-fixed human alveolar (AE) and cystic echinococcosis (CE) liver sections. Scale bars 500µm.
