# Supplementary figures and images for: Identification and characterization of the elusive protein backbone of the immuno-dominant and species-specific Em2(G11) metacestode antigen of Echinococcus multilocularis
Source: Front Parasitol. 2025 Mar 11;4:1540215. doi: 10.3389/fpara.2025.1540215 (PMC11935348; doi:10.3389/fpara.2025.1540215)

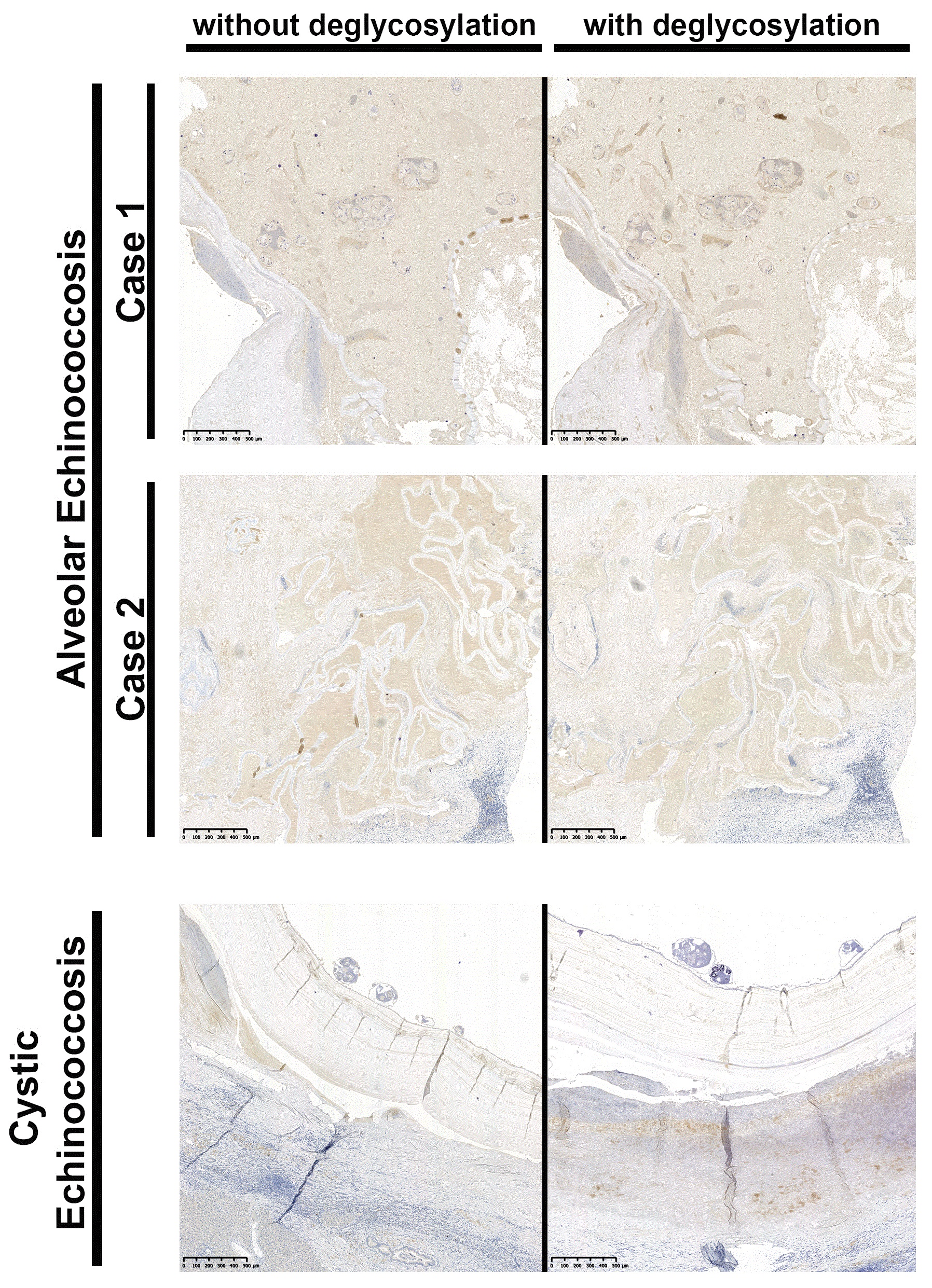

Supplement: Supplementary Figure 1 — IHC-S with control monoclonal antibody mAb D.i 36/1 on deglycosylated and native-fixed human alveolar (AE) and cystic echinococcosis (CE) liver sections. Scale bars 500µm. [file Image1.jpeg]
