# Supplementary material for: Identification and characterization of the elusive protein backbone of the immuno-dominant and species-specific Em2(G11) metacestode antigen of Echinococcus multilocularis
Source: Front Parasitol. 2025 Mar 11;4:1540215. doi: 10.3389/fpara.2025.1540215 (PMC11935348; doi:10.3389/fpara.2025.1540215)
Supplement: Supplementary file 7 [file DataSheet1.docx]

Supplementary Material

# Supplementary Data. Sequence and expression control for the recombinant production of EmuJ_001105600

The approach is described in the Materials and Methods section. The amino acid sequence for EmuJ_001105600 is accessible with the UniProt Accession number A0A068YFE3.

- 1. *Sequence of the expression vector for Em2rec protein.*


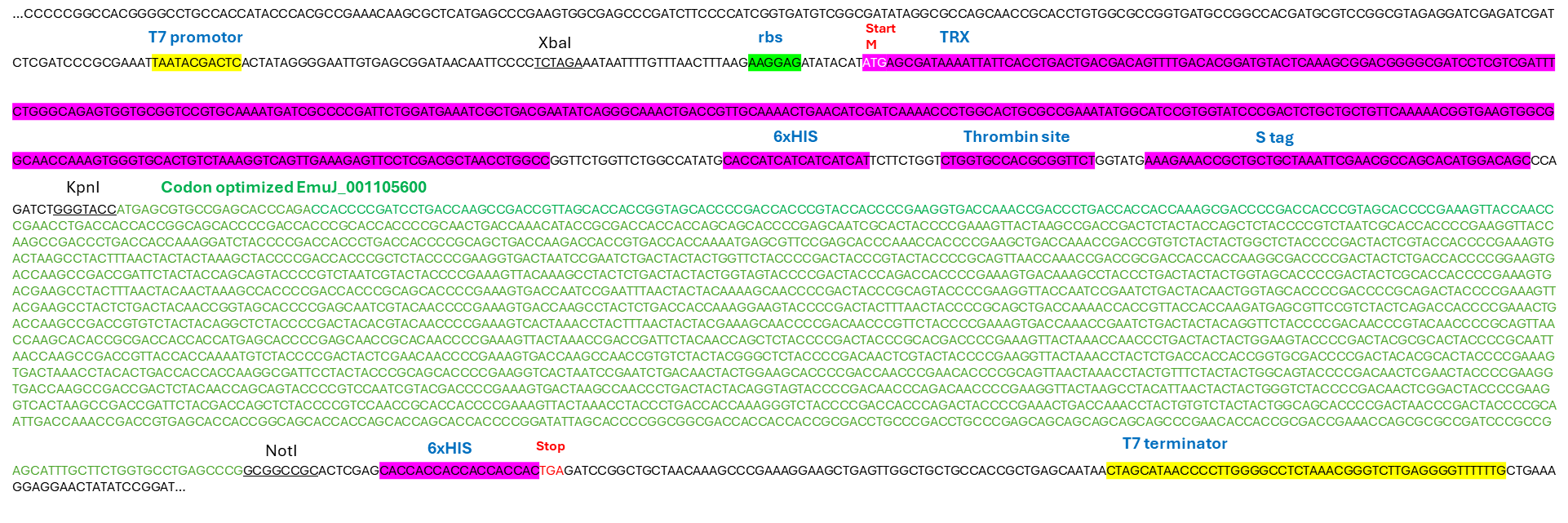
Due to a significantly high content of repetitive sequences, the gene was codon optimized for expression in E. coli, commercially synthesized and cloned into the pET-32α (+) expression vector at the KpnI/NotI restriction sites (GenScript, USA), including a C-terminal poly-His affinity tag (Figure 1). Because of a potential interfering reaction in further approaches, the N-terminal thioredoxin (TRX) tail, thrombin (thr) cleavage site and the S-tag were exchanged with a commercially synthesized 28-bases nonsense fragment (Microsynth, Switzerland) at the XbaI/KpnI restriction sites, while maintaining the ribosome binding site (rbs), having the methionine start-codon (ATG) at the beginning of the gene candidate (Figure 2).

**Figure 1**. Commercial sequence (pet32α(+)-TRX-6xHIS-Thrombin-Stag-**EmuJ_001105600**-6xHIS). The different tags, restriction sites, and the sequence coding for the Em2rec protein are highlighted.

T7promotor (underlined) AATACGACTCACTATAGGGGAATTGTGAGCGGATAACAATTCCCCTCTAGAAATAATTTTGTTTAACTTTAAGAAGGAGATATACATATGAGCGATAAAATTATTCACCTGACTGACGACAGTTTTGACACGGATGTACTCAAAGCGGACGGGGCGATCCTCGTCGATTTCTGGGCAGAGTGGTGCGGTCCGTGCAAAATGATCGCCCCGATTCTGGATGAAATCGCTGACGAATATCAGGGCAAACTGACCGTTGCAAAACTGAACATCGATCAAAACCCTGGCACTGCGCCGAAATATGGCATCCGTGGTATCCCGACTCTGCTGCTGTTCAAAAACGGTGAAGTGGCGGCAACCAAAGTGGGTGCACTGTCTAAAGGTCAGTTGAAAGAGTTCCTCGACGCTAACCTGGCCGGTTCTGGTTCTGGCCATATGCACCATCATCATCATCATTCTTCTGGTCTGGTGCCACGCGGTTCTGGTATGAAAGAAACCGCTGCTGCTAAATTCGAACGCCAGCACATGGACAGCCCAGATCTGGGTACCATGAGCGTGCCGAGCACCCAGACCACCCCGATCCTGACCAAGCCGACCGTTAGCACCACCGGTAGCACCCCGACCACCCGTACCACCCCGAAGGTGACCAAACCGACCCTGACCACCACCAAAGCGACCCCGACCACCCGTAGCACCCCGAAAGTTACCAACCCGAACCTGACCACCACCGGCAGCACCCCGACCACCCGCACCACCCCGCAACTGACCAAACATACCGCGACCACCACCAGCAGCACCCCGAGCAATCGCACTACCCCGAAAGTTACTAAGCCGACCGACTCTACTACCAGCTCTACCCCGTCTAATCGCACCACCCCGAAGGTTACCAAGCCGACCCTGACCACCAAAGGATCTACCCCGACCACCCTGACCACCCCGCAGCTGACCAAGACCACCGTGACCACCAAAATGAGCGTTCCGAGCACCCAAACCACCCCGAAGCTGACCAAACCGACCGTGTCTACTACTGGCTCTACCCCGACTACTCGTACCACCCCGAAAGTGACTAAGCCTACTTTAACTACTACTAAAGCTACCCCGACCACCCGCTCTACCCCGAAGGTGACTAATCCGAATCTGACTACTACTGGTTCTACCCCGACTACCCGTACTACCCCGCAGTTAACCAAACCGACCGCGACCACCACCAAGGCGACCCCGACTACTCTGACCACCCCGGAAGTGACCAAGCCGACCGATTCTACTACCAGCAGTACCCCGTCTAATCGTACTACCCCGAAAGTTACAAAGCCTACTCTGACTACTACTGGTAGTACCCCGACTACCCAGACCACCCCGAAAGTGACAAAGCCTACCCTGACTACTACTGGTAGCACCCCGACTACTCGCACCACCCCGAAAGTGACGAAGCCTACTTTAACTACAACTAAAGCCACCCCGACCACCCGCAGCACCCCGAAAGTGACCAATCCGAATTTAACTACTACAAAAGCAACCCCGACTACCCGCAGTACCCCGAAGGTTACCAATCCGAATCTGACTACAACTGGTAGCACCCCGACCCCGCAGACTACCCCGAAAGTTACGAAGCCTACTCTGACTACAACCGGTAGCACCCCGAGCAATCGTACAACCCCGAAAGTGACCAAGCCTACTCTGACCACCAAAGGAAGTACCCCGACTACTTTAACTACCCCGCAGCTGACCAAAACCACCGTTACCACCAAGATGAGCGTTCCGTCTACTCAGACCACCCCGAAACTGACCAAGCCGACCGTGTCTACTACAGGCTCTACCCCGACTACACGTACAACCCCGAAAGTCACTAAACCTACTTTAACTACTACGAAAGCAACCCCGACAACCCGTTCTACCCCGAAAGTGACCAAACCGAATCTGACTACTACAGGTTCTACCCCGACAACCCGTACAACCCCGCAGTTAACCAAGCACACCGCGACCACCACCATGAGCACCCCGAGCAACCGCACAACCCCGAAAGTTACTAAACCGACCGATTCTACAACCAGCTCTACCCCGACTACCCGCACGACCCCGAAAGTTACTAAACCAACCCTGACTACTACTGGAAGTACCCCGACTACGCGCACTACCCCGCAATTAACCAAGCCGACCGTTACCACCAAAATGTCTACCCCGACTACTCGAACAACCCCGAAAGTGACCAAGCCAACCGTGTCTACTACGGGCTCTACCCCGACAACTCGTACTACCCCGAAGGTTACTAAACCTACTCTGACCACCACCGGTGCGACCCCGACTACACGCACTACCCCGAAAGTGACTAAACCTACACTGACCACCACCAAGGCGATTCCTACTACCCGCAGCACCCCGAAGGTCACTAATCCGAATCTGACAACTACTGGAAGCACCCCGACCAACCCGAACACCCCGCAGTTAACTAAACCTACTGTTTCTACTACTGGCAGTACCCCGACAACTCGAACTACCCCGAAGGTGACCAAGCCGACCGACTCTACAACCAGCAGTACCCCGTCCAATCGTACGACCCCGAAAGTGACTAAGCCAACCCTGACTACTACAGGTAGTACCCCGACAACCCAGACAACCCCGAAGGTTACTAAGCCTACATTAACTACTACTGGGTCTACCCCGACAACTCGGACTACCCCGAAGGTCACTAAGCCGACCGATTCTACGACCAGCTCTACCCCGTCCAACCGCACCACCCCGAAAGTTACTAAACCTACCCTGACCACCAAAGGGTCTACCCCGACCACCCAGACTACCCCGAAACTGACCAAACCTACTGTGTCTACTACTGGCAGCACCCCGACTAACCCGACTACCCCGCAATTGACCAAACCGACCGTGAGCACCACCGGCAGCACCACCAGCACCAGCACCACCCCGGATATTAGCACCCCGGCGGCGACCACCACCACCGCGACCTGCCCGACCTGCCCGAGCAGCAGCAGCAGCAGCCCGAACACCACCGCGACCGAAACCAGCGCGCCGATCCCGCCGAGCATTTGCTTCTGGTGCCTGAGCCCGGCGGCCGCACTCGAGCACCACCACCACCACCACTGAGATCCGGCTGCTAACAAAGCCCGAAAGGAAGCTGAGTTGGCTGCTGCCACCGCTGAGCAATAACTAGCATAACCCCTTGGGGCCTCTAAACGGGTCTTGAG
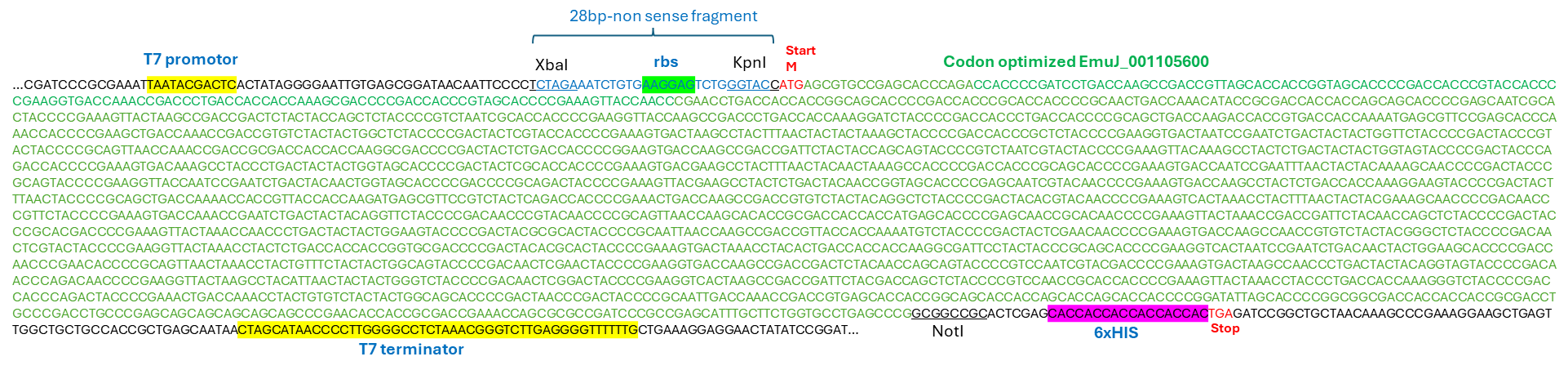
GGGTTTTTTG T7terminator (underlined)

**Figure 2**. Optimized tag-reduced sequence (pet32α(+)-**EmuJ_001105600**-6xHIS).

T7promotor(underlined) AATACGACTCACTATAGGGGAATTGTGAGCGGATAACAATTCCCCTCTAGAAATCTGTGAAGGAGTCTGGGTACCATGAGCGTGCCGAGCACCCAGACCACCCCGATCCTGACCAAGCCGACCGTTAGCACCACCGGTAGCACCCCGACCACCCGTACCACCCCGAAGGTGACCAAACCGACCCTGACCACCACCAAAGCGACCCCGACCACCCGTAGCACCCCGAAAGTTACCAACCCGAACCTGACCACCACCGGCAGCACCCCGACCACCCGCACCACCCCGCAACTGACCAAACATACCGCGACCACCACCAGCAGCACCCCGAGCAATCGCACTACCCCGAAAGTTACTAAGCCGACCGACTCTACTACCAGCTCTACCCCGTCTAATCGCACCACCCCGAAGGTTACCAAGCCGACCCTGACCACCAAAGGATCTACCCCGACCACCCTGACCACCCCGCAGCTGACCAAGACCACCGTGACCACCAAAATGAGCGTTCCGAGCACCCAAACCACCCCGAAGCTGACCAAACCGACCGTGTCTACTACTGGCTCTACCCCGACTACTCGTACCACCCCGAAAGTGACTAAGCCTACTTTAACTACTACTAAAGCTACCCCGACCACCCGCTCTACCCCGAAGGTGACTAATCCGAATCTGACTACTACTGGTTCTACCCCGACTACCCGTACTACCCCGCAGTTAACCAAACCGACCGCGACCACCACCAAGGCGACCCCGACTACTCTGACCACCCCGGAAGTGACCAAGCCGACCGATTCTACTACCAGCAGTACCCCGTCTAATCGTACTACCCCGAAAGTTACAAAGCCTACTCTGACTACTACTGGTAGTACCCCGACTACCCAGACCACCCCGAAAGTGACAAAGCCTACCCTGACTACTACTGGTAGCACCCCGACTACTCGCACCACCCCGAAAGTGACGAAGCCTACTTTAACTACAACTAAAGCCACCCCGACCACCCGCAGCACCCCGAAAGTGACCAATCCGAATTTAACTACTACAAAAGCAACCCCGACTACCCGCAGTACCCCGAAGGTTACCAATCCGAATCTGACTACAACTGGTAGCACCCCGACCCCGCAGACTACCCCGAAAGTTACGAAGCCTACTCTGACTACAACCGGTAGCACCCCGAGCAATCGTACAACCCCGAAAGTGACCAAGCCTACTCTGACCACCAAAGGAAGTACCCCGACTACTTTAACTACCCCGCAGCTGACCAAAACCACCGTTACCACCAAGATGAGCGTTCCGTCTACTCAGACCACCCCGAAACTGACCAAGCCGACCGTGTCTACTACAGGCTCTACCCCGACTACACGTACAACCCCGAAAGTCACTAAACCTACTTTAACTACTACGAAAGCAACCCCGACAACCCGTTCTACCCCGAAAGTGACCAAACCGAATCTGACTACTACAGGTTCTACCCCGACAACCCGTACAACCCCGCAGTTAACCAAGCACACCGCGACCACCACCATGAGCACCCCGAGCAACCGCACAACCCCGAAAGTTACTAAACCGACCGATTCTACAACCAGCTCTACCCCGACTACCCGCACGACCCCGAAAGTTACTAAACCAACCCTGACTACTACTGGAAGTACCCCGACTACGCGCACTACCCCGCAATTAACCAAGCCGACCGTTACCACCAAAATGTCTACCCCGACTACTCGAACAACCCCGAAAGTGACCAAGCCAACCGTGTCTACTACGGGCTCTACCCCGACAACTCGTACTACCCCGAAGGTTACTAAACCTACTCTGACCACCACCGGTGCGACCCCGACTACACGCACTACCCCGAAAGTGACTAAACCTACACTGACCACCACCAAGGCGATTCCTACTACCCGCAGCACCCCGAAGGTCACTAATCCGAATCTGACAACTACTGGAAGCACCCCGACCAACCCGAACACCCCGCAGTTAACTAAACCTACTGTTTCTACTACTGGCAGTACCCCGACAACTCGAACTACCCCGAAGGTGACCAAGCCGACCGACTCTACAACCAGCAGTACCCCGTCCAATCGTACGACCCCGAAAGTGACTAAGCCAACCCTGACTACTACAGGTAGTACCCCGACAACCCAGACAACCCCGAAGGTTACTAAGCCTACATTAACTACTACTGGGTCTACCCCGACAACTCGGACTACCCCGAAGGTCACTAAGCCGACCGATTCTACGACCAGCTCTACCCCGTCCAACCGCACCACCCCGAAAGTTACTAAACCTACCCTGACCACCAAAGGGTCTACCCCGACCACCCAGACTACCCCGAAACTGACCAAACCTACTGTGTCTACTACTGGCAGCACCCCGACTAACCCGACTACCCCGCAATTGACCAAACCGACCGTGAGCACCACCGGCAGCACCACCAGCACCAGCACCACCCCGGATATTAGCACCCCGGCGGCGACCACCACCACCGCGACCTGCCCGACCTGCCCGAGCAGCAGCAGCAGCAGCCCGAACACCACCGCGACCGAAACCAGCGCGCCGATCCCGCCGAGCATTTGCTTCTGGTGCCTGAGCCCGGCGGCCGCACTCGAGCACCACCACCACCACCACTGAGATCCGGCTGCTAACAAAGCCCGAAAGGAAGCTGAGTTGGCTGCTGCCACCGCTGAGCAATAACTAGCATAACCCCTTGGGGCCTCTAAACGGGTCTTGAGGGGTTTTTTG T7terminator (underlined)

- 1. *Pilot protein expression control of Em2rec protein*


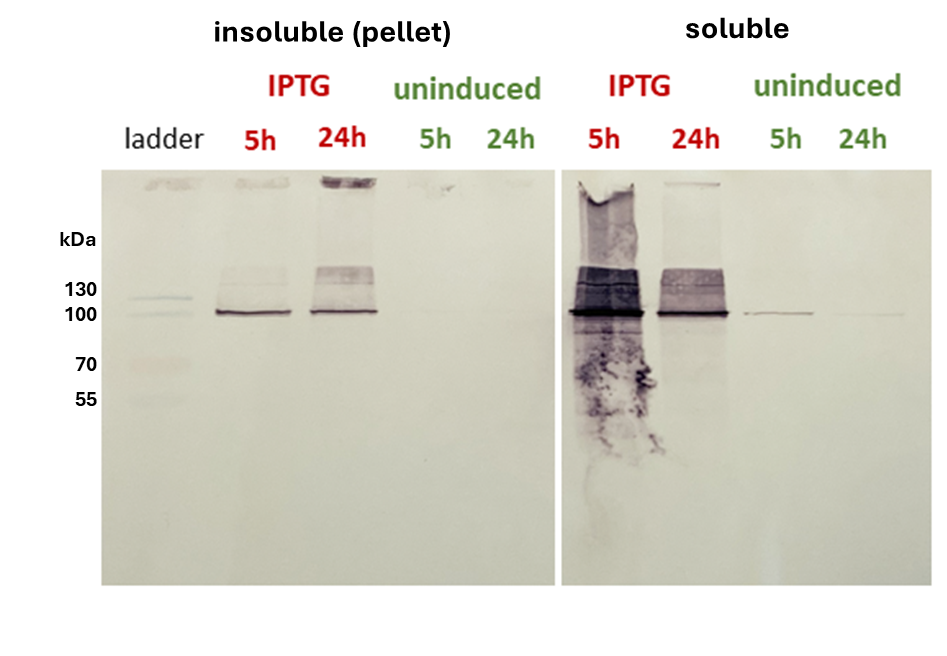


**Figure 3**. Pilot expression control of Em2rec with 0.5 mM IPTG. To confirm the production of the 92-kDa protein, 1 mL of a bacteria culture (in 5 mL culture media) was centrifuged after 5 and 24 hours, and the pellet was lysed. The soluble (supernatant) and insoluble (pellet) protein fractions were analysed by Western blot using an anti-His monoclonal antibody (MA1-21315-BTIN, ThermoFisher Scientific, USA), a Streptavidin-AP conjugate (BioRad #1703554) followed by incubation with a 1-Step™ NBT/BCIP Substrate Solution (ThermoScientific # 34042).

- 1. *Mass production and protein purification by Immobilized Metal Affinity Chromatography*

A mass production of Em2rec with 500 mL of bacterial culture media (LB) was performed induced at an OD600 of 0.6 with 0.5 mM IPTG for 6 hours at 26 °C. The media was centrifuged and the pellet lysed to purify the soluble protein by a His-Trap HP column on an Äkta pure M1 FPLC (Cytiva, USA).


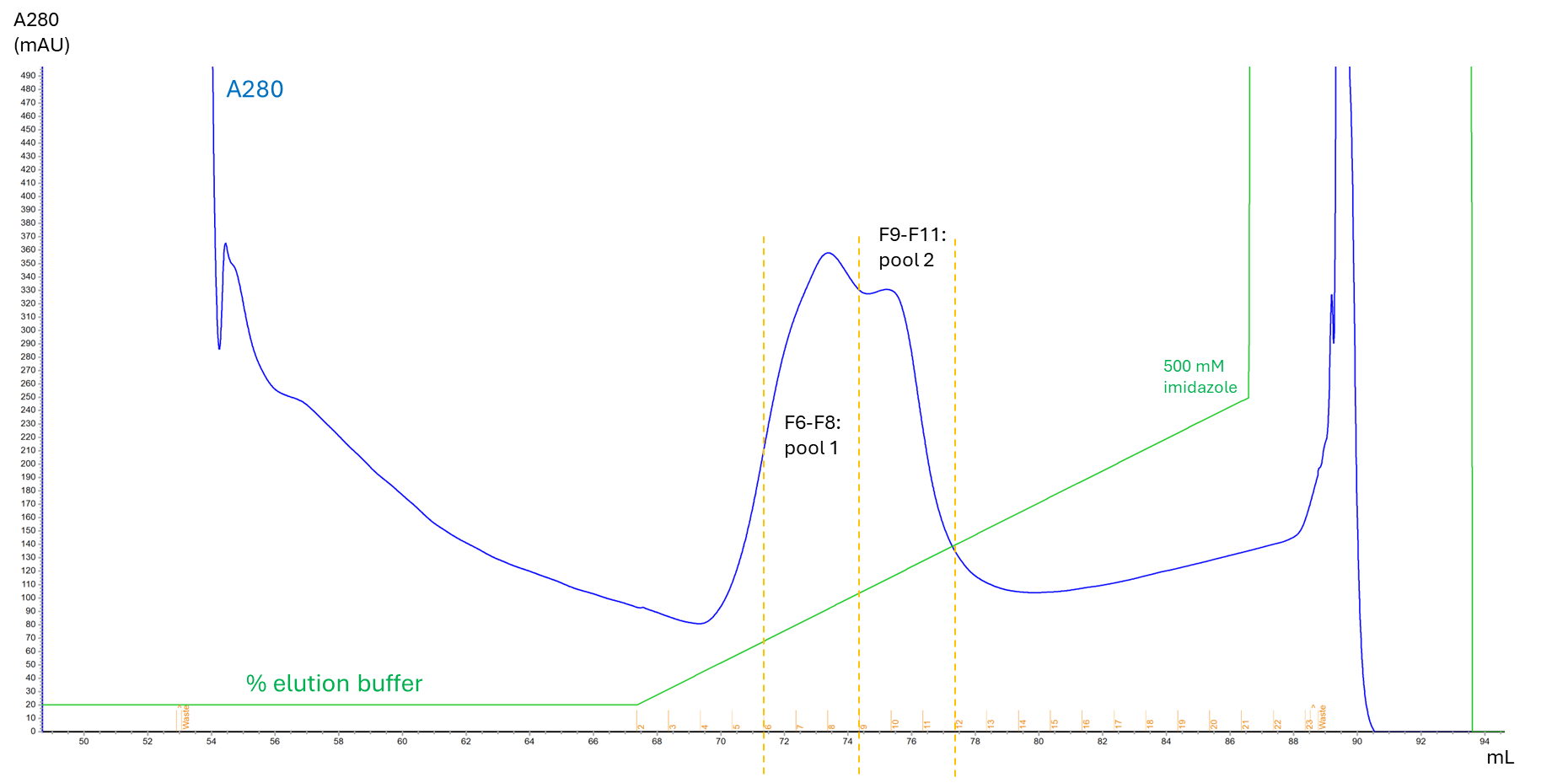


**Figure 4**. Immobilized Metal Affinity Chromatography (IMAC) with a 1 mL His-Trap HP column (GE29-0510-21, Cytiva, USA) on an Äkta pure M1 FPLC (Cytiva, USA) eluted by an increasing concentration of imidazole (40-500 mM). Eluted fractions that contained protein were pooled, buffer exchanged to phosphate buffered saline (PBS) and concentrated to a volume of 0.5 mL using a 10 kDa MWCO Amicon® Ultra centrifugation filter (UFC8010, Merck, Germany). The protein concentration by Pierce BCA Protein Assay was 1.1 mg/mL and 0.61 mg/mL for pool 1 and pool 2, respectively, with a purity of > 80% (Figure 3).


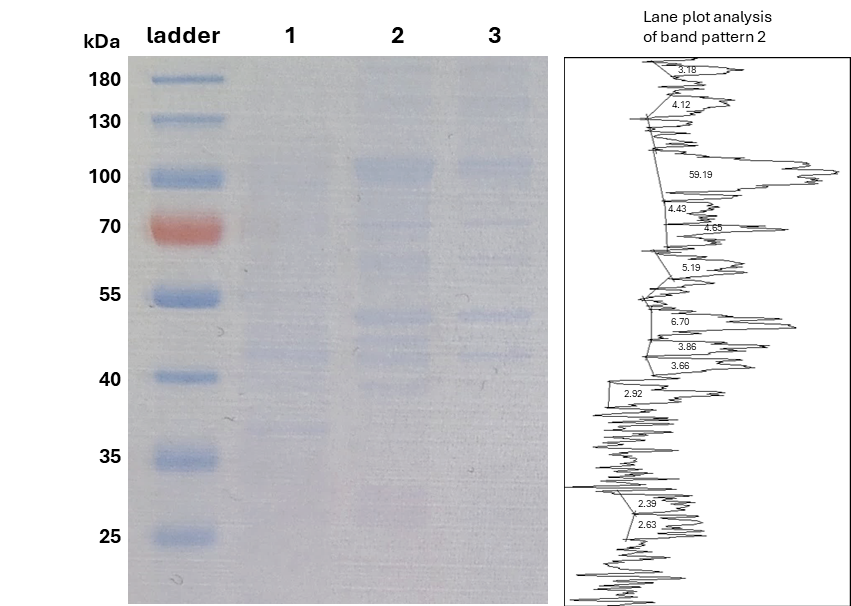


**Figure 5**. SDS-PAGE analysis of the IMAC purified Em2rec. Lane 1: unbound flow-through of the IMAC. Lane2: elution pool 1. Lane 3: elution pool 2. The purity of the Em2rec at around 100 kDa was estimated by lane plot analysis of the lane 2 in ImageJ, which was around 60%, and > 60% considering higher molecular weight bands potentially being aggregates (see Figure 3).
